# Phylogeny of C_4_-Photosynthesis Enzymes Based on Algal Transcriptomic and Genomic Data Supports an Archaeal/Proteobacterial Origin and Multiple Duplication for Most C_4_-Related Genes

**DOI:** 10.1371/journal.pone.0110154

**Published:** 2014-10-14

**Authors:** Shan Chi, Shuangxiu Wu, Jun Yu, Xumin Wang, Xuexi Tang, Tao Liu

**Affiliations:** 1 Ocean University of China, Qingdao, Shandong Province, People’s Republic of China; 2 CAS Key Laboratory of Genome Sciences and Information, Beijing Key Laboratory of Genome and Precision Medicine Technologies, Beijing Institute of Genomics, Chinese Academy of Sciences, Beijing, P. R. China; 3 Beijing Key Laboratory of Functional Genomics for Dao-di Herbs, Beijing Institute of Genomics, Chinese Academy of Sciences, Beijing, China; The National Orchid Conservation Center of China; The Orchid Conservation & Research Center of Shenzhen, China

## Abstract

Both Calvin-Benson-Bassham (C_3_) and Hatch-Slack (C_4_) cycles are most important autotrophic CO_2_ fixation pathways on today’s Earth. C_3_ cycle is believed to be originated from cyanobacterial endosymbiosis. However, studies on evolution of different biochemical variants of C_4_ photosynthesis are limited to tracheophytes and origins of C_4_-cycle genes are not clear till now. Our comprehensive analyses on bioinformatics and phylogenetics of novel transcriptomic sequencing data of 21 rhodophytes and 19 Phaeophyceae marine species and public genomic data of more algae, tracheophytes, cyanobacteria, proteobacteria and archaea revealed the origin and evolution of C_4_ cycle-related genes. Almost all of C_4_-related genes were annotated in extensive algal lineages with proteobacterial or archaeal origins, except for phosphoenolpyruvate carboxykinase (*PCK*) and aspartate aminotransferase (*AST*) with both cyanobacterial and archaeal/proteobacterial origin. Notably, cyanobacteria may not possess complete C_4_ pathway because of the flawed annotation of pyruvate orthophosphate dikinase (*PPDK*) genes in public data. Most C_4_ cycle-related genes endured duplication and gave rise to functional differentiation and adaptation in different algal lineages. C_4_-related genes of NAD-ME (NAD-malic enzyme) and PCK subtypes exist in most algae and may be primitive ones, while NADP-ME (NADP-malic enzyme) subtype genes might evolve from NAD-ME subtype by gene duplication in chlorophytes and tracheophytes.

## Introduction

The Calvin-Benson-Bassham (CBB) cycle is the most important autotrophic CO_2_ fixation pathway on today’s Earth, and widely distributes among all land plants, algae and cyanobacteria [1]. The characteristic enzyme involved in CBB cycle is ribulose 1,5-bisphosphate carboxylase/oxygenase (RubisCO), which catalyzes the primary carboxylation of ribulose 1,5-bisphosphate (RuBP) and yields two molecules of 3-phosphoglycerate (PGA), a C_3_ compound [2]. Therefore, the CBB cycle is also called C_3_ cycle [2]. Numerous reviews have summarized the understandings of this important pathway [2], [3].

Another important CO_2_ fixation pathway is Hatch-Slack (H-S) cycle, found in sugarcane in 1965 [4]. It is also well known as C_4_ cycle because, in counterpart with the C_3_ cycle, the first enzyme involved in this cycle is phosphoenolpyruvate carboxylase (PEPC), which catalyzes the primary carboxylation of phosphoenolpyruvate (PEP) to form a 4-carbon acid compound oxaloacetate (OAA) as the first photosynthetic product. As compared with C_3_ plants, the C_4_ cycle develops novel and efficient CO_2_ concentration mechanisms, on anatomical and biochemical function, to enhance RubisCO performance even at limited ambient CO_2_ levels and result in significant decreases in photorespiration, improvement of photosynthetic efficiency and water use efficiency during CO_2_ fixation [5]. Thus, C_4_ plants are capable of growing in habitats that may be too harsh for C_3_ species, such as rock outcrops and hypersaline or arid soils of low latitude, and contribute about a quarter of the primary productivity on the planet [6].

For land plants, C_4_ plants can be divided into three distinct biochemical variants based on different decarboxylation modes: NADP-malic enzyme (NADP-ME) type, NAD-malic enzyme (NAD-ME) type, and phosphoenolpyruvate carboxykinase (PCK) type [7]. Each C_4_ subtype consists of two shared enzymes (PEPC and PPDK) and two or three other unique enzymes of their own, which are all encoded by nuclear genome and transported to different position of cell to catalyze corresponding reaction.

Through the phylogenetic studies in species of *Flaveria* (Asteraceae) and *Brassica gravinae*, C_4_ plants are believed to have evolved gradually from C_3_ plants through several intermediate stages of C_3_–C_4_ plants [8]. However, C_4_-cycle genes and pathway analysis are mostly limited to land plants till recently. It is widely accepted that land plants evolved from streptophyte algae (a diverse group of green, fresh water algae) [9]. Algae are the principal primary producers in oceanic and freshwater communities, and also are responsible for the net flux of about 2 gigatons of carbon per year from the atmosphere to the lithosphere [10]. Furthermore, some C_4_-cycle genes and intermediates were found in a few algal species [11]–[13], although the presence of the whole pathway was not confirmed in algae. Therefore the phylogenetic analysis of C_4_ pathway-related (C_4_-related) genes using recently released algal genomic and transcriptomic data has important scientific values for understanding the origin and evolution of photosynthesis.

With the development of the new generation sequencing (NGS) technology, more algal genome and transcriptome sequencing data were released (Table S1, including their references), including several species of marine phytoplankton such as diatoms *Thalassiosira pseudonana* and *Phaeodactylum tricornutum*, green algae *Ostreococcus tauri* and *Micromonas*, brown alga *Ectocarpus siliculosus*, and red algae *Pyropia haitanensis* and *Pyropia yezoensis*. In addition, as a part of the recent 1000 Plant (OneKP) Project (http://www.onekp.com), we provided 19 marine phaeophytes and 21 marine rhodophytes for transcriptome sequencing. These new released algal sequencing data provide us more valuable gene information and more extensive algal lineages to screen the key genes in algae. Therefore, in this study, by analyzing these sequencing data, we confirmed the existence of C_4_-related genes in extensive algal lineages. We further resolved to perform a comprehensive phylogenetic analysis using a much larger dataset (especially including much more diverse algal lineages, land plants, cyanobacteria, γ-proteobacteria and archaea) to elucidate the evolution of C_4_-related genes and possible pathway types. Our analyses, for the first time, support a non-cyanobacterial origin for almost all of the C_4_-related genes, with the exception of phosphoenolpyruvate carboxykinase gene (*PCK*) and aspartate aminotransferase (*AST*), which have both cyanobacterial endosymbiont and non-cyanobacterial origin. This result is quite different from the previous study on C_3_ photosynthesis origin from a cyanobacterial primary endosymbiont.

## Results

### Sequencing yield and annotation of unigenes

The transciptomic sequencing of 21 red and 19 brown algal species yielded a total of 503,310,608 raw reads, equal to the length of 89.2 Gb with an average sequence length of 180 bases. The reads were assembled into 2,161,986 scaffolds, with an average length of 717 bp and an N50 of 1751 bp. All sequences were aligned against the local nr protein database downloaded from NCBI using the BLASTx algorithm for gene annotation. When the E-value cutoff was set at 10^−5^, a total of 585,247 unigenes had significant BLAST matches.

### KEGG pathway analysis of CO_2_ fixation genes in diverse algal lineages

**Figure 1:**
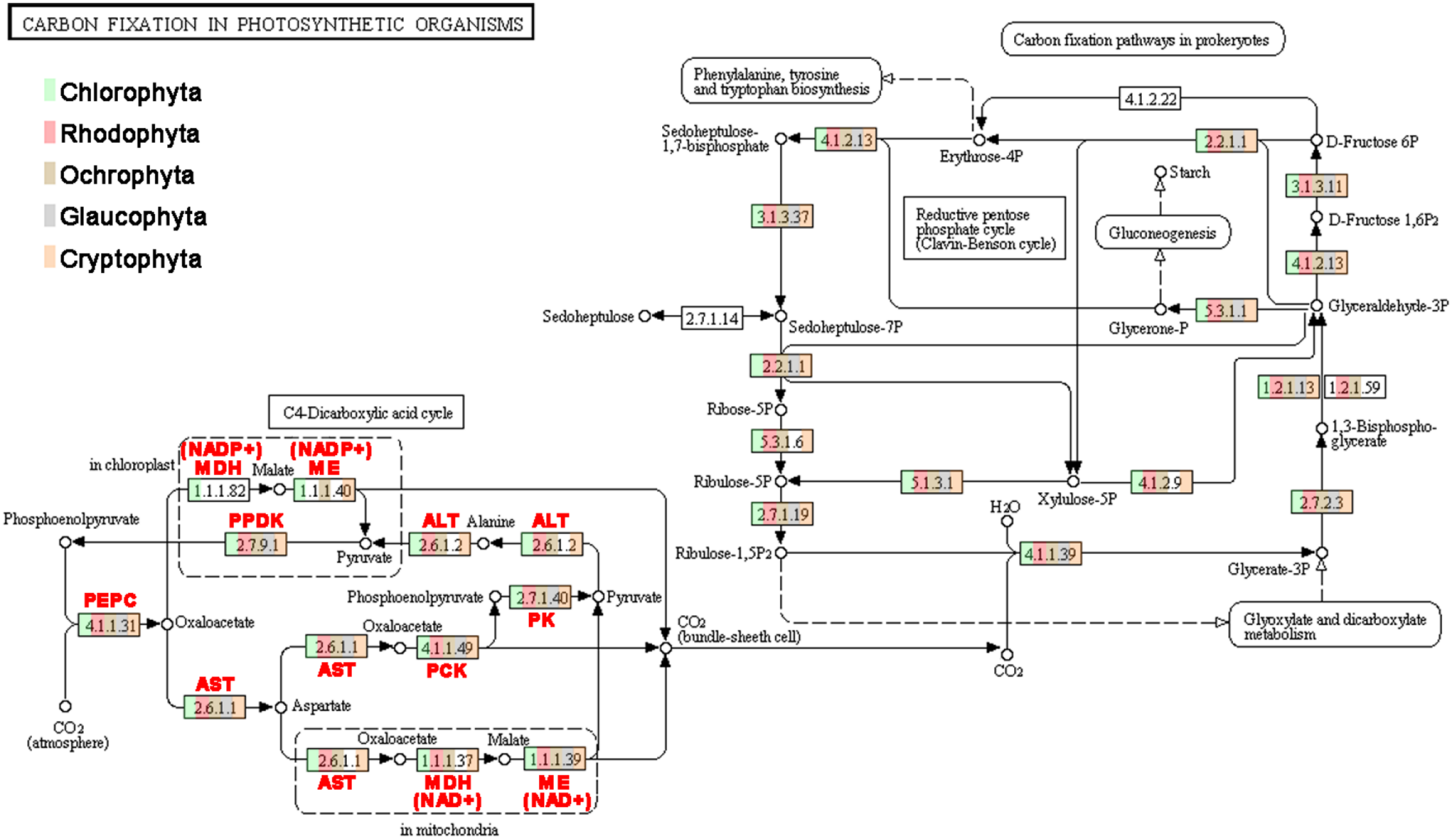
Genes identified for coding enzymes in CO_2_ fixation pathways in algal species generated by KEGG. Different colors in the small boxes represent the genes identified from Rhodophyta, Ochrophyta, Chlorophyta, Glaucophyta and Cryptophta, respectively. The numbers within the small boxes are the enzyme commission (EC) number. PEPC: phosphoenolpyruvate carboxylase; PPDK: pyruvate, orthophosphate dikinase; ALT: alanine transaminase; MDH: malate dehydrogenase; ME: malic enzyme; AST: aspartate aminotransferase; PK: pyruvate kinase; PCK: phosphoenolpyruvate carboxykinase.

These transcriptomic data were further performed on KEGG pathway analysis. We also included genomic data from 19 sequenced algae (Table S1) for a thorough identification of genes encoding enzymes related to the carbon fixation pathways in detail and all enzymes in C_3_ and C_4_ pathways among diverse algal groups (Chlorophyta, Rhodophyta, Ochrophyta, Glaucophyta and Cryptophyta) (Fig. 1). The numbers of gene in C_3_ and C_4_ pathways in algal species and some representative tracheophytes are listed in Table 1. Our results provide an unequivocal molecular evidence that most of the C_3_-pathway and C_4_-pathway genes are actively transcribed in these algal groups and demonstrate the possibility of the extensive existence of different photosynthetic pathways in algae.

**Table 1:** The numbers of gene in C_3_ and C_4_ pathways in algae and tracheophytes species.

Taxonomy	Organism	*ALT*	*AST*	*MDH*	*ME*	*PCK*	*PEPC*	*PK*	*PPDK*
Chlorophyta	*Bathycoccus prasinos*								1
Chlorophyta	*Chlamydomonas reinhardtii*	2	5	4	2	1	2	2	2
Chlorophyta	*Chlorella variabilis*					2	2	2	
Chlorophyta	*Coccomyxa subellipsoidea*	2							
Chlorophyta	*Micromonas pusilla*						1		
Chlorophyta	*Micromonas sp.*	1							
Chlorophyta	*Ostreococcus lucimarinus*	1	2	2	3		1		
Chlorophyta	*Ostreococcus tauri*		2		2		1	1	1
Chlorophyta	*Volvox carteri f nagariensis*	2	2	2	1	2	1	2	1
Cryptophyta	*Guillardia theta*	2	7	3	6	1	2	2	1
Glaucophyta	*Cyanophora paradoxa*		4	4		1	1	1	
Ochrophyta	*Aureococcus anophagefferens*	1						2	
Ochrophyta	*Nannochloropsis gaditana*		5	1	2	2	1	5	
Ochrophyta	*Nannochloropsis oceanica*								
Ochrophyta	*Colpomenia sinuosa*	1	2	2	1	1	1	3	
Ochrophyta	*Desmarestia viridis*	1	3	1	2	1		3	1
Ochrophyta	*Dictyopteris undulata*	1	2	2	2			3	1
Ochrophyta	*Ectocarpus siliculosus*	1	3	2	2	1	1	3	1
Ochrophyta	*Ishige okamurae*			1	1	1		3	1
Ochrophyta	*Petalonia fascia*	1	3	1	2	1		2	
Ochrophyta	*Phaeodactylum tricornutum*	1	4	1	2	1	2	2	
Ochrophyta	*Punctaria latifolia*	1	3	2	3	1		3	
Ochrophyta	*Saccharina japonica*	1	2	2	1	1		2	
Ochrophyta	*Saccharina sculpera*	1	3	2	2	1			
Ochrophyta	*Sargassum fusiforme*	1	2	1	1				
Ochrophyta	*Sargassum hemiphyllum var. chinense*	1	3	2		1		3	1
Ochrophyta	*Sargassum henslowianum*	1	2	1	1	1	1	2	1
Ochrophyta	*Sargassum horneri*	1	2	2	2	1	1	2	1
Ochrophyta	*Sargassum integerrimum*	1	3	1	1	1	1	2	1
Ochrophyta	*Sargassum muticum*	1	2	2	2	1			
Ochrophyta	*Sargassum thunbergii*	1	4	2	1	1	1	2	1
Ochrophyta	*Sargassum vachellianum*	1	3	2	2	1	1	3	1
Ochrophyta	*Scytosiphon dotyi*	1	2	2	2	1		3	
Ochrophyta	*Scytosiphon lomentaria*	1	3	2	1	1	1	3	1
Ochrophyta	*Thalassiosira oceanica*	3	1	1		1			
Ochrophyta	*Thalassiosira pseudonana*		3	2	1	1	1		1
Ochrophyta	*Undaria pinnatifida*	1	4	2	1	1		2	
Rhodophyta	*Ahnfeltiopsis flabelliformis*	1	1	1	1		1	2	1
Rhodophyta	*Betaphycus philippinensis*	1	1	1	1		1	2	1
Rhodophyta	*Ceramium kondoi*	1		2	1			2	
Rhodophyta	*Chondrus crispus*		2	1	2		1	2	
Rhodophyta	*Cyanidioschyzon merolae*	1	2	2	1		1	3	
Rhodophyta	*Dumontia simplex*	1	1	1	1		1	2	1
Rhodophyta	*Eucheuma denticulatum*	1	2	1	2		1	2	1
Rhodophyta	*Galdieria sulphuraria*	1	3	3	1	2	1	4	
Rhodophyta	*Gloiopeltis furcata*	1	1		2			2	
Rhodophyta	*Gracilaria blodgettii*	1	1	1	1		1	2	1
Rhodophyta	*Gracilaria chouae*	1		1	1		1	2	1
Rhodophyta	*Gracilaria vermiculophylla*	1	2	1	1		1	2	1
Rhodophyta	*Gracilariopsis lemaneiformis*	1	1	2	1		1	2	1
Rhodophyta	*Grateloupia catenata*	1	1	1	1		1	2	1
Rhodophyta	*Grateloupia chiangii*		1	1	1		1	2	1
Rhodophyta	*Grateloupia filicina*		1				1		
Rhodophyta	*Grateloupia livida*	1	1		1		1	1	
Rhodophyta	*Grateloupia turuturu*	1	2		1		1	2	1
Rhodophyta	*Heterosiphonia pulchra*	1		1	1			1	1
Rhodophyta	*Kappaphycus alvarezii*		1		1			1	
Rhodophyta	*Mazzaella japonica*		1	3	2		1	2	
Rhodophyta	*Neosiphonia japonica*	1	1	1	2		1	1	1
Rhodophyta	*Pyropia yezoensis*		3	1		1	1	2	
Rhodophyta	*Symphyocladia latiuscula*	1	1	2	1		1	2	1
Tracheophytes	*Arabidopsis thaliana*	2	6	11	6	4	4	13	1
Tracheophytes	*Oryza sativa*	2	6	10	7	4	6	10	4
Tracheophytes	*Sorghum bicolor*	1	3	7	11	3	4	11	3
Tracheophytes	*Zea mays*	9	5	2	1	1	3	11	1
Bryophytes	*Physcomitrella patens*	8	3	11	4	4	7	15	5

### Phylogenetic analysis of C_4_-related genes

**Figure 2:**
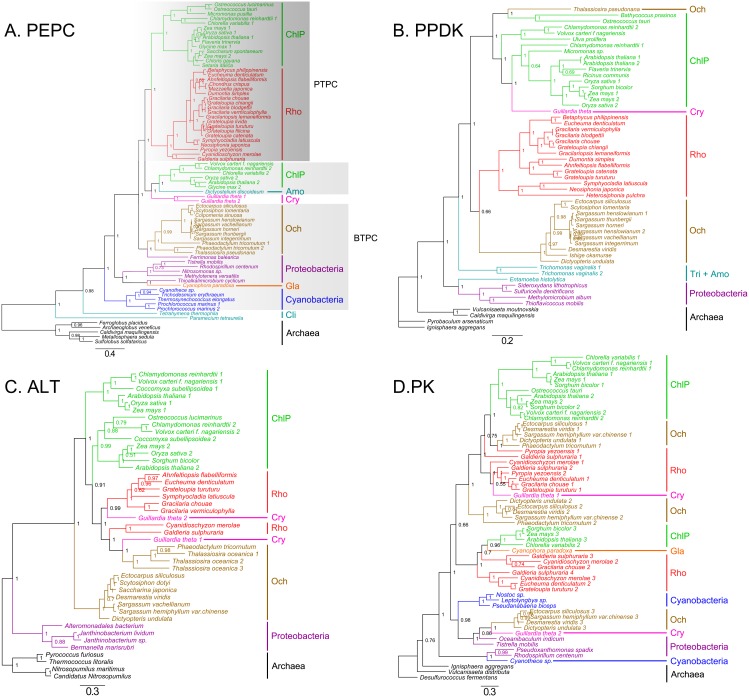
Bayesian phylogenetic trees of PEPC (A) PPDK (B), ALT (C) and PK (D) enzymes with bootstrap values (when >50%) indicated at the nodes. The PEPCs are classified as PTPCs (in dark gray boxes) and BTPCs (in light gray boxes). ChlP, chlorophytes and plants. Gla, glaucophytes. Rho, rhodophytes. Och, ochrophytes. Cry, cryptophytes. Tri, trichomonad. Amo, amoeba. Cil, ciliates.

**Figure 3:**
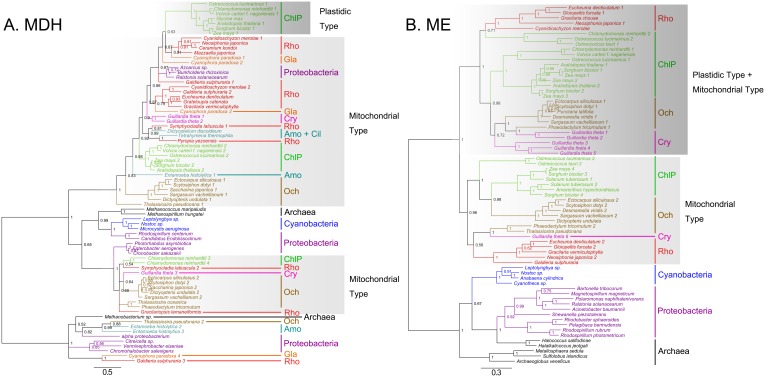
Bayesian phylogenetic trees of MDH (A) and ME (B) enzymes with bootstrap values (when >50%) indicated at the nodes. The plastic type MDHs are in dark gray boxes, and mitochondrial types in light gray boxes. Eukaryotic MEs cluster in two clades, one clade in dark gray boxes consists of both plastic and mitochondrial type, the other one only contains mitochondrial type. ChlP, chlorophytes and plants. Gla, glaucophytes. Rho, rhodophytes. Och, ochrophytes. Cry, cryptophytes. Tri, trichomonad. Amo, amoeba. Cil, ciliates.

**Figure 4:**
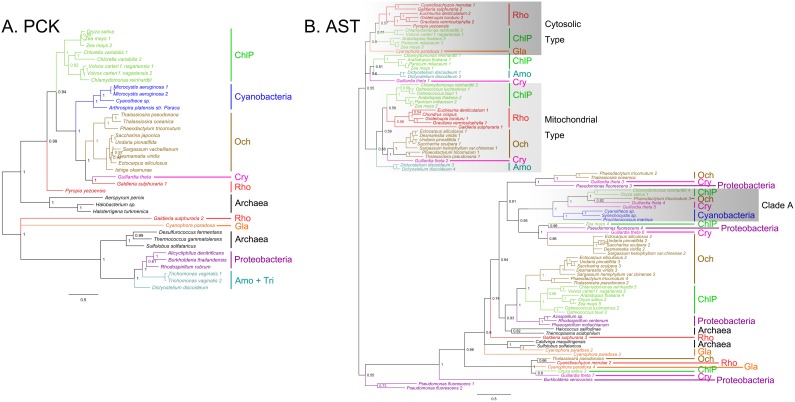
Bayesian phylogenetic trees of PCK (A) and AST (B) enzymes with bootstrap values (when >50%) indicated at the nodes. The cytosolic types of AST are in dark gray boxes and mitochondrial types are in light gray boxes. Besides this two clades, some chlorophyte, plant and cryptophyte ASTs are grouped with cyanobacteria (Clade A), which are also in light gray boxes. ChlP, chlorophytes and plants. Gla, glaucophytes. Rho, rhodophytes. Och, ochrophytes. Cry, cryptophytes. Tri, trichomonad. Amo, amoeba. Cil, ciliates.

In addition of diverse algal species and tracheophytes, wealth of candidate C_4_ gene sequences (Table S2) were also detected among archaea, proteobacteria and cyanobacteria. Therefore, we built phylogenetic trees that display relationships of full amino acid sequences of C_4_ related genes from archaea, proteobacteria, cyanobacteria, tracheophytes, and algae based on Bayesian method (only representative candidates are included to save space). The results show that the eukaryotic C_4_ enzymes have an archaeal/proteobacterial core (Fig. 2, 3, and 4). The phylogenetic trees of *PEPC* (phosphoenolpyruvate carboxylase), *PPDK* (pyruvate, orthophosphate dikinase), *ALT* (alanine transaminase), *MDH* (malate dehydrogenase), *ME* (malic enzyme), and *PK* (pyruvate kinase) (see Figs. 2, 3, and 4) support their non-cyanobacterial origin in primary endosymbiotic algae. However, almost all eukaryotic *PCK*s have a cyanobacterial origin through endosymbiosis gene transfer (EGT). Some genes of ochrophytes and cryptophytes potentially have a red algal origin (e.g., *ME*) as expected under the secondary endosymbiosis hypothesis, and others have archaeal/proteobacterial origins inherited from their endosymbiosis host genomes or acquired from non-cyanobacterial archaea or proteobacteria via horizontal gene transfer (HGT) (e.g., *PEPC*).

### *PEPC* originated from archaea/proteobacteria and evolved into diverse types in different eukaryotic organisms

PEPC (EC 4.1.1.31) is an important enzyme for CO_2_ fixation in the C_4_-cycle and shared by three subtypes of C_4_-pathway metabolisms. The enzyme is present in almost all plants, green algae, cyanobacteria, most archaea, and non-photosynthetic bacteria, but is absent from animals and fungi [14]. In this study, we, for the first time, find its homologous sequences widely exist in various algae species, including chlorophytes, ochrophytes, rhodophytes, glaucophytes and cryptophytes (Fig. 1).

There are two homologous of *PEPC* genes in chlorophytes and tracheophytes, termed as bacterial-type (BTPC) and plant-type (PTPC) according to their amino acid sequences and structures [15]. Especially, the deduced PEPC polypeptides are readily classified as a BTPC or PTPC based on their C-terminal tetrapeptide, which is either (R/K) NTG for BTPCs or QNTG for PTPCs [16]. We compared the full deduced sequence of PEPCs among all above five algal groups (chlorophytes, ochrophytes, rhodophytes, glaucophytes and cryptophytes), cyanobacteria, proteobacteria and archaea. The results show that rhodophytes only possess PTPCs, whereas ochrophytes, glaucophytes, proteobacteria, and cyanobacteria only contain BTPCs. PEPCs of archaea, cryptophytes and ciliates do not have the typical C-terminal tetrapeptide and can not be classified into any known types.

**Figure 5:**
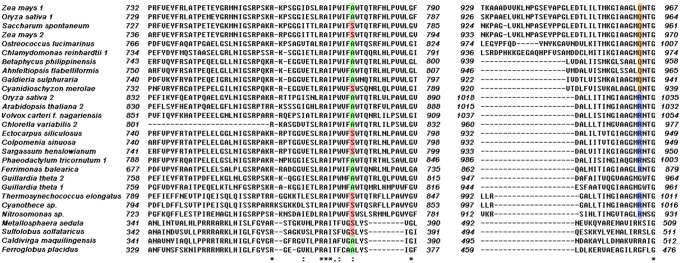
Alignments of partial amino acid sequences of PEPC isoenzymes between species of Rhodophyta, Ochrophyta, chlorophytes, Cryptophta, bacteria and archaea. The deduced PEPC sequences were aligned using ClustalX 1.83 software. Numbering indicates the position of the first and last residue in each aligned sequence. Semi-colons and asterisks indicate identical and conserved amino acids respectively. The C-terminal tetrapeptide (R/K)NTG for BTPCs is highlighted in blue color, whereas QNTG for PTPCs is highlighted in orange. The amino acid residue of No. 774 or around it is highlighted in green (non-photosynthetic PEPCs) or red (C4-type PEPCs).

Phylogenic analysis suggests that *PEPC* broadly distributes among algae and plants and is likely to have a non-cyanobacteria origin. Within the strict consensus tree, archaeal PEPCs is the first group to diverge at the base of the tree, followed by ciliates clade, BTPCs clusters, cryptophytes clade, and a large group including PTPCs of chlorophytes and rhodophytes which form a single well-resolved clade in two clusters with strong support (Bayesian posterior probability, PP = 0.98) (Fig. 2A). This topology suggests that BTPCs maybe more primitive than PTPCs and the latter one presumably have arisen from the BTPC homologs. Furthermore, PTPC can be classified into C_3_-type, C_3_-like-type, C_3_–C_4_ intermediate type, and C_4_-type according to the sequence context (the amino acid residue at 774 or those around it at the C-end of PEPCs) [17]. All C_4_-type PTPCs investigated to date harbor a serine (S) residue at the corresponding position but this very site is replaced by an alanine (A) residue in all non-photosynthetic PEPCs. Site-specific mutagenesis studies demonstrated that this amino acid residue plays a key role in enzyme kinetics [18]. In addition, we found that the differentiation between non-C_4_-type and C_4_-type is not limited in PTPCs but also happens in BTPCs (Fig. 5). According to the criteria mentioned above, BTPCs of chlorophytes and tracheophytes are identified as non-C_4_-type, whereas in ochrophytes, diatoms possess non-C_4_-type site and Phaeophyceae algae prefer to C_4_-type. Interestingly, fractions of cyanobacteria and archaea also have the C_4_-type site (Fig. 5).

### *PPDK*, *ALT*, and *PK* have a non-cyanobacteria archaea/proteobacteria origin

**Figure 6:**
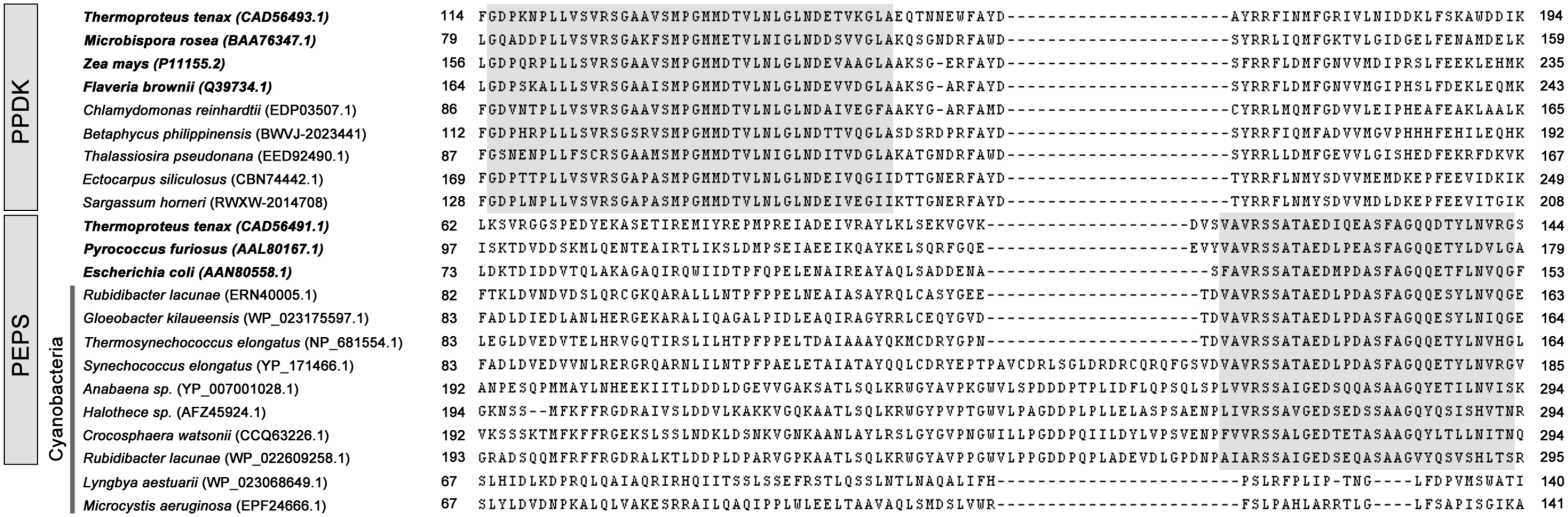
Partial sequence alignment of the N-terminal domains of PEPS and PPDK enzymes. Numbering indicates the position of the first and last residue in each aligned sequence. Proteins with biochemically confirmed enzyme activity are indicated by gray shading. Grey shading indicate the identified sequence signatures specific for PEPS and PPDK respectively.

The enzyme PPDK (EC 2.7.9.1), another shared enzyme in the C_4_ cycle (Fig. 1), catalyzes the reversible phosphorylation of pyruvate and inorganic phosphate yielding P-enolpyruvate and inorganic pyrophosphate at the expense of a single molecule of ATP [19]. Using present gene data in NCBI, *PPDK* homologs have not been detected in the available cyanobacterial genomes. Actually we find that all published cyanobacterial *PPDK* are not real *PPDK*, for they lack the *PPDK* N-terminal nucleotide-binding domain. Instead, they should be classified as other PEP-utilizing genes, such as phosphoenolpyruvate synthetase (*PEPS*; pyruvate, water dikinase) (Fig. 6).

In the consensus tree of *PPDK* (Fig. 2B), archaeal sequences also cluster at the base like the PEPC tree, neighbored by clades of proteobacteria and protists, indicating its origin from archaea/proteobacteria. A separate clade is formed by rhodophytes with moderate bootstrap support, while Phaeophyceae algae (Ochrophyta) cluster independently and appear as the sister taxon of rhodophytes. Tracheophytes, chlorophytes, diatoms, and cryptophytes form another large clade. Therefore, primary endosymbiotic algal *PPDK* gene seems originated from archaea or proteobacteria, while Phaeophyceae algae possibly inherit the gene from red algal endosymbionts.

Similar to *PPDK*, neither dose *ALT* have any cyanobacterial homolog, indicating the eukaryotic *ALT* is also non-cyanobacterium-derived gene (Fig. 2C). Cryptophytes and diatoms formed a well supported clade with rhodophytes, which indicates their revolution relationship with red algae endosymbionts.

The analysis of *PK* genes is much more complicated due to its enormous duplications in eukaryotes. Our phylogenetic analysis also excluded the cyanobacterial origin of *PK* in algae and tracheophytes (Fig. 2D). Rhodophytes, glaucophytes, chlorophytes, and tracheophytes obtained this gene from archaea/proteobacteria, and gene duplication occurred extensively at least twice in these species. Cryptophytes and Phaeophyceae algae have two types of *PK*, one is inherited from red algae through EGT (eg. *Guillardia theta* 1, EKX52520.1), another is transferred from some proteobacteria or archaea (eg. *Guillardia theta* 2, EKX43540.1). The latter type is not found in diatoms, and this absence suggests different evolutionary pattern, such as gene lost, between Phaeophyceae algae and diatoms.

Note that Sogin and Zillig *et al*. theorized that the eukaryotic nucleus arose from the cellular fusion between both a bacterium and an archaeon [20]. Lang *et al*. indicated several hundred eukaryotes genes are acquired from the mitochondrial ancestor derived from an endosymbiotic alpha-proteobacterium that was engulfed by a eukaryotic- or archaebacteria-like cell more than one billion years ago [21]. Therefore, we suggest that the archaea/proteobacteria-derived C_4_-related genes in eukaryotic organisms may inherit from the endosymbiotic host genome, which had acquired numerous genes from bacterium and archaeon or transferred from archaea/proteobacteria through HGT.

### *MDH* and *ME* originated from archaea/proteobacteria and evolved into different homologs after duplication

The phylogenic analysis of *MDH* and *ME* has encountered difficulties from sequence similarity between NADP- and NAD-dependent homologs. Therefore, we built phylogenies separately for *MDH* and *ME*, albeit they probably shared a common ancestry (Fig. 3). According to the phylogenetic tree, it is clear that eukaryotic *MDH*s are considered essentially archaeal/proteobacterial in provenance (Fig. 3A). Targeting signal predictions for plastid and mitochondrion genes in algae and plants suggest that the gene encoding plastid-targeted *MDH* in tracheophytes and chlorophytes likely evolved from eukaryotic algal mitochondrial homologs via gene duplication and acquisition of a plastid-import signal. Ochrophyte *MDH* has two separate origins: one from red algae endosymbionts (eg. *Saccharina japonica* 2, KM113483), the other from secondary endosymbiotic hosts or archaea/proteobacteria through HGT (eg. *Saccharina japonica* 1, KM113482). Cryptophytes only acquired *MDH* from red algal endosymbionts (Fig. 3A).

The strict *ME* consensus tree displays a relatively simple topology compared to the *MDH* tree. It shows that the *ME* genes are also obtained very early in algal evolution but start to duplicate before the emergence of primary endosymbiotic algae, might be in its last eukaryotic common ancestor. After duplication, some Archaeplastida (including red algae, green algae and plants) obtained two homologs of *ME* from archaea/proteobacteria, and these genes are likely to be transmitted into ochrophytes and cryptophytes by EGT from their red algal endosymbionts (Fig. 3B). Targeting signal predictions suggest two clades with different organellar locations. One clade is composed of plastid target genes and the other consists of both plastid and mitochondrion target genes.

### *PCK* and *AST* originated from both cyanobacteria and archaea/proteobacteria

The remaining C_4_ genes, *PCK* and *AST*, have a complex evolutionary history. Our phylogenetic analysis shows they may have arisen from multiple origins (Fig. 4A). For instance, two copies of *PCK*s from *Galdieria sulphuraria* cluster into separate clades: one is related to archaea and proteobacteria (eg. *Galdieria sulphuraria* 2, EME28834.1), the other groups with cyanobacteria (eg. *Galdieria sulphuraria* 1, EME27660.1). Therefore, rhodophytes may acquire *PCK* from two different ancestors. In contrast, glaucophytes possess *PCK*s of archaea/proteobacteria origin. Ochrophytes and cryptophytes may obtain *PCKs* from red algal endosymbionts.

The evolution of eukaryotic *AST* genes appears much more complicated. In our phylogenetic tree, the enzymes clustered into two separate clades. One clade consists of cytosolic and mitochondrial *AST*s. In the constricted tree (Fig. 4B), mitochondrial *AST* is present in tracheophytes, chlorophyte, rhodophyte, ochrophyte, and cryptophyte algae, and the cytoplasmic *AST* is present in archaeplastida (Rhodophyta, Virideplantae and Glaucophyta) and forms a monophyletic group without any secondary endosymbiotic algae. These species all group with homologs of proteobacteria, suggesting their proteobacteria origin. However interestingly, in our phylogenetic tree, in addition to the mitochondrial and cytoplasmic types, *AST*s of some chlorophyte and cryptophyte algae are grouped into another clade (clade A) with cyanobacteria, indicating their cyanobacteria endosymbiont origin.

## Discussion

### C_4_ genes distributed among diverse algae mostly with archaeal/proteobacterial origins

Our new data allow the identification of almost all the enzymes necessary for C_4_ photosynthesis widely distributing among different eukaryotic algal lineages (Fig. 1). Furthermore, we find that most of eukaryotic C_4_ genes have an archaeal/proteobacterial core but algal *PCK*s and *AST*s originated from both cyanobacteria and archaea/proteobacteria, based on our phylogenetic analyses by using diverse genes from archaea, proteobacteria, cyanobacteria, tracheophytes and various algae groups (Fig. 2, 3, 4).

Notably, we found that cyanobacteria do not possess complete C_4_ pathway. Though *PEPC*s are proved existed in cyanobacteria (Fig. 2A), the cyanobacterial *PPDK* sequences published in GenBank are excluded from our analysis (Fig. 2B), because we for the first time found that the published cyanobacterial *PPDK* sequences should actually be annotated as *PEPS* or other PEP-utilizing genes because of the lacking of the *PPDK* N-terminal nucleotide-binding domain [22]. Neither are *ALT* and *PK* found their homologs in cyanobacteria (Fig. 2C, D), suggesting that the C_4_ cycle dose not arisen from a cyanobacterial endosymbiosis. Our further phylogenic analysis strongly supports this hypothesis that almost all of the C_4_-related genes have a non-cyanobacteria origin (Fig. 2B).

We also searched the cyanobacterium-like plastid (also termed as cyanelle) genome of Glaucophyta *Cyanophora paradoxa*, which is considered as a “living fossil” and a paradigm for the invasion of a eukaryotic cell by a cyanobacterium [23], but did not find any complete C_4_ pathway, except for partial sequences of *PPDk* and *ALT* homologs (data not shown). As nucleomorphs are considered as the enslaved red or green algal nucleus residuals, we further searched the nucleomorph genomes of Cryptophyta *Guillardia theta* (AF165818.4, AJ010592.2, AF083031.2), *Hemiselmis andersenii* (CP000881.1, CP000882.1, CP000883.1), *Cryptomonas paramecium* (CP002172.1, CP002173.1, CP002174.1) and Cercozoa *Bigelowiella natans* (DQ158856.1, DQ158857.1, DQ158858.1), and did not find any C_4_ genes, neither. The results are consistent with our hypothesis that the C_4_ cycle is not evolved from cyanobacterial-endosymbiotic event.

### C_4_-related genes of diverse subtypes in different algal lineages

In land plants, C_4_ photosynthesis are typically divided into three subtypes-NADP-ME, NAD-ME, and PCK-based on different decarboxylation models. Different C_4_ species often use one of the three subtypes nearly exclusively [24]. Some experimental results suggest that the PCK subtype is maximal in biomass production and CO_2_ fixation [25], [26], and others show that the NAD-ME and PCK subtypes are more adaptive than the NADP-ME subtype for species in arid environments [24].

By surveying a selection of cyanobacterial and algal species to determine the gene composition (Table S3), we found all chlorophytes own all three varieties of C_4_-related genes, just like their derived lineage, tracheophytes. However, virtually all species of rhodophytes and ochrophytes, including diatoms which have been proved to be able to incorporate CO_2_ into the C-4 carboxyl of C_4_ acids [11], own NAD-ME and PCK subtypes genes, but lack the *MDH* (NADP) of NADP-ME subtype. This result suggests that diatoms, red algae and brown algae quite possibly have similar C_4_-related genes, and NADP-ME type C_4_ genes may emerge in chlorophytes after they separated from other algal lineages.

In tracheophytes, the cytosolic and mitochondrial AST activities are connected by C_4_ photosynthesis and they both participate in the NAD-ME subtype of C_4_ pathway, while cytosolic *AST* itself can function as the PCK type [27]. In our analysis, Archaeplastida all evolved to have both cytosolic *AST* and mitochondrial *AST* except that glaucophytes only have cytosolic *AST* (Fig. 4B). In addition, no known C_4_ cytosolic *AST* homolog has been annotated in ochrophytes and cryptophytes, but *AST*s of these secondary endosymbiotic algae are grouped into another clade with proteobacteria/archaea (Fig. 4B), indicating different evolution of C_4_-related genes between primary and secondary endosymbiotic algae.

### Gene duplication of C_4_-related enzymes

Our phylogenic analyses also suggest that most C_4_-related genes undergo gene duplications subsequently at different evolutionary time scale (Fig. 2, 3, 4). Furthermore, there are diverse gene duplications among different eukaryotic lineages. For instance, there are two types of *PEPC* termed as PTPC and BTPC in chlorophytes and tracheophytes, which demonstrate that the *PEPC* gene duplication had already occurred in the ancestral chlorophytes, with BTPCs being the ancestral type.

Our study showed that the primary endosymbiotic algae evolved to have both cytosolic *AST* and mitochondrial *AST* except glaucophytes may only have cytosolic *AST* (Fig. 4B), suggesting *AST* duplicated and functional differentiated in a common algal ancestor before the primary endosymbiosis, and the absence of mitochondrial *AST* in glaucophytes may be due to gene lost. Another group of *AST* found in the secondary endosymbiotic algae, including diatoms, Phaeophyceae and cryptophytes (Fig. 4B), may also be the results of gene lost and functional differentiation after gene duplication which happened in their common ancestor before the secondary endosymbiosis.

Gene duplication and evolution in *MDH* and *ME* are even more fascinating. NADP-dependent and NAD-dependent *MDH*s of the two separate C_4_ subtypes seem to share the same ancestor, and duplication took place in the last common ancestor of chlorophytes and tracheophytes (Fig. 3A). The same event happened during evolution of *ME*, yet duplication maybe occurred earlier in the last eukaryotic common ancestor (Fig. 3B). The ancestral mitochondrial enzymes endured duplication and gave rise to homologs which evolved plastid target signal peptide. These plastid *MDH* and *ME* genes finally took part in the construction of NADP-ME type C_4_ pathway in tracheophytes.

Since gene duplication is a necessary contribution to genetic novelty and adaptation and requirement [28], the multiple copies of C_4_-related genes should have arisen accompanying the emergence of different C_4_ cycle which made the regulation more delicate during the evolution of C_4_ photosynthesis.

### Origin and evolution of C_4_ pathway

Our analysis suggests that the C_4_-related genes (except for *PCK* and *AST*) in eukaryotic organisms originated from archaea/proteobacteria. It is widely accepted that the eukaryotic nucleus may have arisen from the cellular fusion between either a bacterium or “protoeukaryote” and an archaeon [20]. Therefore, a substantial number of archaea/proteobacteria-derived genes are retained in the eukaryotic nuclear genome. Another mechanism for algae and plants acquiring archaea/proteobacteria genes is horizontal gene transfer (HGT) from archaea or proteobacteria that occurred throughout the history of eukaryotes [29]. Thus, the archaea/proteobacteria-derived C_4_-related genes may inherit from the endosymbiotic host genome or transferred from archaea/proteobacteria through HGT.

Previous study showed that photosynthetic eukaryotes (i.e., algae and plants) gained C_3_ photosynthesis from a cyanobacterial primary endosymbiont [30]. The eukaryotic C_3_ pathway consists of eleven enzymes, and molecular phylogenetic analyses show that recruitment of single enzymes from different pathways could be the driving force for C_3_ pathway evolution in chlorophytes, tracheophytes and rhodophytes [31], [32]. Therefore, we would like to propose a hypothesis on C_4_ cycle formation.

First, when a cyanobacterium was engulfed and retained by a heterotrophic eukaryote, its *PCK* and *AST* genes transferred into the host nucleus and were inherited by the primary endosymbiotic algae to lay out the main framework of PCK subtype of C_4_ pathway with some archaea/proteobacteria-derived C_4_ genes founding in plants.

In addition to the PCK subtype, NAD-ME subtype was also constructed as the result of a patchwork assembly in tracheophytes, but it is difficult to predict which one is more primitive. However, the present work proved that NADP-ME subtype should be a derivant of NAD-ME subtype according to our phylogenic analysis of *MDH* and *ME* genes. The plastid *MDH*s in chlorophytes and tracheophytes are originated from its mitochondrial homologs via duplication (Fig. 3A), and a clade contains both mitochondrial and plastid *ME* homologs are originated from mitochondrial ones (Fig. 3B).

The changing CO_2_ concentration may also be a major environmental driving force for eukaryotes to develop C_4_ metabolism, to suppress photorespiration. Throughout Earth’s geological history, eukaryotes had been exposed to much higher CO_2_ at the beginning of evolutional history but then became starved by a steep decrease of CO_2_ and increase of O_2_ as the outcome of appearance of C_3_ photosynthesis. Some plants can dramatically change the photosynthetic and anatomical traits to meet different environment pressure. For example, *Eleocharis vivipara*, an amphibious sedge, changes its photosynthetic pathway from C_3_ to C_4_ under conditions of CO_2_ deficiency [33]. So did in aquatic environments, in order to reduce the impact of increasing oxygen pressures, aquatic photosynthetic organisms evolved special mechanisms to efficiently maintain carbon fixation. It has been demonstrated that many aquatic photosynthetic organisms can take up both CO_2_ and HCO^3−^ from the surrounding media and this capacity is greatly strengthened under CO_2_-limiting conditions, which is generally known as the inorganic carbon-concentrating mechanism (CCM) [34].

For algae, it is believed that the C_3_ cycle is predominant in the CO_2_ fixation pathway [35], as a result of the endosymbiotic acquisition of a cyanobacterium that evolved into the chloroplasts [31]. However, the existence of a C_4_ photosynthetic pathway in algae or marine phytoplankton is a long-standing debate [36]. Recent papers have reported the evidence for the operation of C_4_ photosynthesis as an alternative CCM in the marine diatom *Thalassiosira weissflogii*, using ^14^C-labeling experiments [11], [12]. In addition, some intermediate products of the C_4_ pathway had been detected in brown alga *Ascophyllum nodosum*, euglenoid *Euglena gracilis*, and dinoflagellates [13], and C_4_-like photosynthetic characteristics had also been detected in green alga *Udotea flabellum* [37]. Until recently, few studies are focused on C_4_-cycle genes in limited algae species, and imperfect detection method in algae may bring about ambiguous results. Our current work provides molecular evidences for further biochemical and physiological experiment validation on more algal species to prove the existence of C_4_-related genes, or even C_4_ cycle in some algal species or extensive algal lineages.

## Materials and Methods

### Ethics Statement

Marine brown and red algal samples were collected along the coast of China during October, 2010 to March, 2012, and were provided by Culture Collection of Seaweed at the Ocean University of China. The location is neither privately owned nor protected places. No specific permissions were required for these locations, and the study did not involve any endangered or protected species. The species information and the GPS coordinates of their specific locations are provided in Table S4.

### RNA extraction

Total RNA was extracted from algal tissues using an improved CTAB method for brown algal samples and an improved Trizol method for red algal samples, and RNA quantity and quality was examined as previous reports [38], [39].

### Transcriptome sequencing and de novo assembly

cDNA library construction and sequencing were performed by the BGI (Shenzhen, China) on Illumina (San Diego, USA) HiSeq instruments in accordance with the manufacturer’s instructions. Strict reads filtering was performed before *de novo* assembly using SOAP denovo-Trans (http://soap.genomics.org.cn/SOAPdenovo-Trans.html). Pair-end reads with primer or adaptor sequences were removed. Reads with more than 10% of the bases below Q20 quality or more than 5% of unknown nucleotides (Ns) were filtered from total reads. Gapcloser was further used for gap filling of the scaffolds.

### C_4_-cycle gene identification and KEGG pathway analysis

The assembled sequences were BLASTx against the nr protein database downloaded from NCBI with E-value<10^−5^. Deduced algal C_4_ protein coding sequences were further examined for their homology by using BLAST X and BLAST P available at NCBI. These sequences were uploaded to GenBank database, and their accession numbers were listed in Table S2. All other publicly available C_4_ enzymes sequences were obtained from GenBank databases (Table S2). The accession numbers of gene in C3 and C4 pathways in tracheophytes species were listed in Table S5. To reconstruct the metabolic pathways, high-quality assembled algal sequences and 19 sequenced algal genome sequences downloaded from GenBank database (Table S1) were assigned to KEGG Automatic Annotation Server (www.genome.jp/tools/kaas/) to provide functional annotation of genes.

### Sequence alignments and phylogenetic analyses

Sequences were aligned with ClustalX 1.83 software before Bayesian analysis using MrBayes 3.1.2 software. Analyses were performed as two independent runs, each with four incrementally heated Metropolis-coupled Monte-Carlo Markov Chains running for 5,000,000 generations. Trees were sampled every 100 generations. A total of 50,001 trees in one files were read and the first 25% of trees were discarded as the burn-in. The average standard deviation of split frequencies at the end of the run was below 0.01, indicating stationary conditions.

### Cellular target prediction

The probability of plastid or mitochondrion targeting was assessed according to the on-line prediction service at CBS (http://www.cbs.dtu.dk/services/) with the ChloroP V1.1 and TargetP V1.1 servers using the default values.

## Supporting Information

Table S1 Update information of publicly available algal genome sequences.
(XLSX)

Table S2 Taxonomical list of taxa included in this study. The sequences used to reconstruct phylogenetic trees are written in bold letters, the other Rhodophyta and Ochrophyta full-length sequnces in regular letters are also obtained in present study. Sequence data of Cyanophora paradoxa are acquired from Cyanophora Genome Database.
(XLSX)

Table S3 Genes identified for coding enzymes of each biochemical variant of C_4_ photosynthesis in cyanobacteria and algae species.
(XLSX)

Table S4 Species information of 18 brown algae and 21 red algae for transcriptome sequencing.
(XLSX)

Table S5 The accession numbers of gene in C_3_ and C_4_ pathways in tracheophytes species.
(XLSX)
